# Comparison Between Two Methodologies of Sample Preservation for RNA Extraction in Naturally Delivered Ovine Placenta

**DOI:** 10.3390/ani15060786

**Published:** 2025-03-10

**Authors:** Florencia Aránguiz, Javiera Bahamonde, Francisco Sales, Matías Araya, César Ulloa-Leal, Marcelo Ratto, Camila Sandoval

**Affiliations:** 1 Facultad de Ciencias Veterinarias, Universidad Austral de Chile, Valdivia 5090000, Chile; florencia.aranguiz@alumnos.uach.cl (F.A.); javiera.bahamonde@uach.cl (J.B.); marceloratto@uach.cl (M.R.); 2 Instituto de Investigaciones Agropecuarias, Centro Regional de Investigación INIA Kampenaike, Punta Arenas 6200000, Chile; fsales@inia.cl; 3 Facultad de Ciencias Veterinarias y Pecuarias, Universidad de Chile, Santiago 8320000, Chile; matias.araya.b@ug.uchile.cl; 4 Escuela de Ciencias Agrícolas y Veterinarias, Universidad Viña del Mar, Viña del Mar 2520000, Chile; cesar.ulloa@uvm.cl

**Keywords:** delivered placenta, sheep, RNA

## Abstract

Placental samples for RNA extraction are collected via non-recovery (euthanasia) or invasive (surgery) methods in small ruminants, such as sheep. Alternatively, delivered placentas could be used, but the feasibility of obtaining high-quality RNA from this tissue is unknown in sheep. We aimed to evaluate the possibility of extracting RNA from naturally delivered ovine placenta, comparing two preservation methods. Twenty-seven single-pregnant sheep were monitored 24/7 from gestational day 140 to parturition. Tissue was collected after placental delivery, preserved using snap frozen (SF, *n* = 27) and RNAlater^®^ (LTR, *n* = 27) techniques, and processed for RNA extraction using a commercial kit. RNA concentration (ng/µL), A260/280, and RNA quality number (RQN) were measured. Concentration was higher (*p* < 0.001) in LTR (70.39 ± 6.3) than in SF (49.77 ± 10.5), A260/280 was higher (*p* = 0.045) in SF (2.06 ± 0.01) than in LTR (2.03 ± 0.01), and RQN was higher (*p* < 0.0001) in SF (6.81 ± 0.24) than in LTR (2.84 ± 0.24) samples. Timing of placental delivery did not affect the evaluated indicators. Results indicate that extracting high-quality RNA from delivered placentas preserved via the snap-frozen technique is possible, supporting a method that aligns with the refinement principle of animals used in research.

## Simple Summary

The study of the placenta is relevant due to its central role in reproduction. Evaluating gene expression is a valuable tool to assess placental function. To accomplish this, tissue samples are used to extract RNA, a molecule whose quality is essential for successful gene expression studies. A challenge in small ruminants, such as sheep, is that samples are collected postmortem (non-recovery) or surgically (invasive). An alternative could be to use naturally delivered placenta. However, the placenta is rich in enzymes that degrade RNA, being unknown if postpartum sheep placenta will be suitable for RNA extraction. We evaluated if high-quality RNA could be obtained from delivered ovine placenta and compared two preservation methods: 1. snap frozen (SF, samples preserved using liquid nitrogen) and 2. RNAlater^®^ (LTR, samples submerged in RNA preserving solution). Placental expulsion timing has great variety among sheep, so we evaluated if this has an impact on RNA quantity and quality. Our major findings indicate that it is possible to extract RNA from naturally delivered placenta, but RNA quality was acceptable only when using the SF method. We also found that the timing of placental delivery did not affect the quality of RNA.

## 1. Introduction

The study and comprehension of placental development, function and adaptation to different environmental conditions are essential in the field of reproductive physiology. Placenta is a transitory organ that provides an interface for nutrient and gas exchange between the fetus and the dam. It is also an endocrine organ that secretes key hormones for pregnancy maintenance, such as progesterone and placental lactogen [1,2]. Given its central role in the success of mammalian reproduction, collecting high quality samples for the assessment of this organ is of great interest for the research community in this field. However, this is also challenging, as placental tissue rapidly degrades after parturition [3], and collecting samples can be difficult when animal experiments are conducted under field conditions.

For ruminant reproductive physiology, the study of how the placenta responds to different environmental or nutrition managements is of high relevance in livestock raised under extensive conditions, because understanding this has a direct impact on animal production [4]. Additionally, small ruminants such as sheep are valuable models for translational research [5,6], even though there are placental differences between species. Anatomically, sheep and human placentas differ. The sheep placenta is called cotyledonary, and it has several discrete fetal-maternal attachment units (placentomes) which are formed by the fetal side (cotyledon) and the maternal side (caruncle) which is a non-glandular part of the uterine wall. The human placenta has one fetal-maternal contact unit which is a large discoid structure. In terms of invasiveness, the sheep placenta is synepitheliochorial with binucleate cells that represent a fusion between trophoblast and uterine epithelium. The human placenta is classified as hemochorial, which is more invasive because maternal blood is in direct contact with the chorion (fetal portion of placenta). Regardless of the differences, the discoid structure of the human placenta is also structured into cotyledons whose villous tree are structurally similar to the ones in the sheep placenta [7]. Because of those vascular similarities, and because of offspring are born with a similar level of maturity in humans and sheep, the latter is a valuable model for translational research in placental development and fetal studies.

Among the different laboratory techniques for placental assessment, transcriptomics appears as a powerful tool that provides valuable information regarding gene expression, allowing for rapid advances in the understanding of placental physiology [8]. However, one of the most limiting aspects of performing transcriptomics analysis is the obtention of high-quality, undegraded RNA. The level of RNA degradation is measured through parameters such as RNA quality number (RQN), which is a value that ranges from 1 to 10, being 1 the worst quality indicating a fully degraded molecule, and 10 the best quality, or intact RNA [9]. This value is obtained using a Fragment Analyzer system. It is based on the analysis of an electropherogram which in undegraded samples shows two clear peaks, associated with the 18S and 28S ribosomal subunits, respectively, which normally appear in undegraded eukaryotic RNA [10]. The RQN value is calculated based on the area under the 18S and 28S peaks, and the ratio between the peaks. This value is equivalent to RNA integrity number (RIN), which is more broadly used, and it is also based on an electropherogram analysis but performed in a Bioanalyzer System [11]. Values of RQN above 7 are representative of well-preserved and high-quality RNA, which is suitable for analysis such as qPCR, RNA-seq, or other transcriptomic techniques [12]. RNA integrity is the most important parameter to consider when performing transcriptomics analysis because a degraded molecule will lead to erroneous results [13,14].

Accordingly, most of the literature indicates that placental samples must be collected and preserved as soon as possible to avoid RNA degradation because this tissue is enriched in RNase activity [15,16], RNA is labile outside the organism, and its integrity largely determines the precision of transcriptomic analysis [3,17]. Hence, the sampling techniques for placental tissues in ruminants, particularly small ruminants such as sheep, are primarily postmortem via euthanasia [18,19,20], or through surgical extraction of placentomes [21]. This is, in part, due to the unknown impact that collecting a sample from a naturally delivered placenta may have on RNA quantity and quality, and the risk of contamination that would likely occur under field conditions when working with livestock animals. However, nowadays the societal concerns about ethics when using animals for research are increasing, and procedures such as euthanasia or invasive surgeries are being questioned by a rising part of the general public, and debated within scientific groups [22]. Hence, it would be ideal to have other alternatives available to collect tissue when aiming to perform transcriptomics placental studies in sheep.

An alternative collection method could be tissue preservation immediately postpartum after natural placental expulsion, avoiding environmental contamination of the sample. However, placental delivery timing in sheep is variable and may take up to 6 h [23], and the RNA integrity could be affected within that window of time, but this is currently unknown for this species. To our knowledge, there is a lack of literature regarding RNA quantity and quality parameters and also for different sample preservation methods for naturally delivered sheep placenta. The gold standard preservation method is freezing tissues in liquid nitrogen (snap frozen or flash frozen) [24]. However, the appearance of RNA preservation solutions, such as RNAlater^®^, provides a preserving method that can be used to collect samples at room temperature, which may be more convenient when working in field conditions. Currently, there is no information regarding which of these two preservation alternatives may give better results when working with naturally expelled ovine placenta. Hence, our aims were to evaluate if it is possible to extract RNA from naturally delivered placentas in sheep, to compare the effect of two different preservation methods (snap frozen versus RNA preservation solution) on placental RNA quantity and quality, and to evaluate if variation in placental expulsion timing has an impact on RNA quality and quantity parameters.

We aim to evaluate a sample collection approach that reaches a balance between quality research results and the application of the refinement principle of the three Rs approach (reduction, refinement, and replacement) for animals used in research. This principle refers to reducing the invasiveness of procedures applied to animals as much as possible without severely affecting research results [25,26].

## 2. Materials and Methods

### 2.1. Animals

The study was carried out at the Regional Research Center INIA Kampenaike, located in Magallanes, Chile (Lat 52°36′; Lon 70°56′). A total of 55 Corriedale sheep of similar age (3 years old), weight (54.05 ± 0.78 kg), and body condition score (2.39 ± 0.09 in a 1 to 5 scale) were selected for the study. Ewes were divided into five groups for estrus synchronization using two injections of prostaglandin F2α (Ciclase^®^, Zoetis, Parsippany-Troy Hills, NJ, USA) administered 12 days apart. The synchronization protocol was applied to each of the five groups, leaving 1 day between each group. The purpose of this was to alleviate the expected number of parturitions per day to facilitate the post-delivery placental sampling. Ewes were artificially inseminated and pregnancy diagnosis via ultrasound scan was performed on gestational day (GD) 70. Dams carrying singleton pregnancies (*n* = 27) were selected for the present study. These animals and procedures were part of another ongoing study and were also used for this manuscript in order to maximize the scientific benefit of the applied protocols. Animals were maintained under natural grazing conditions during all pregnancies (CP 6.1%, ME 1.6 Mcal/Kg, stocking rate 0.9 ewes/hectare; DM 525 kg/hectare). On GD 140, animals were moved into 1.2 m^2^ individual pens inside a barn. Sheep remained there until 1 day after parturition to monitor the health status of the dam and its lamb. The pens allowed the animals to see each other. The barn had a slotted floor and was covered with a washable and permeable net, minimizing urine or other fluid contamination. Each animal received ad libitum water and was fed with hay and concentrate twice a day according to their nutritional requirements. The floor of each pen was cleaned daily to avoid accumulation of feces or hay. It was also cleaned once an animal showed early signs of parturition. Animals were under 24/7 surveillance by trained personnel from GD 140 until 1 day after parturition.

### 2.2. Placental Sampling and Preservation

Placentas were collected by trained personnel immediately after delivery. It was retrieved when still hanging from the vulva of the ewe, only if it could be extracted by minimal traction. In a few cases, the placenta was collected from the floor immediately after being delivered. The time between parturition and placental delivery was recorded for each animal. The entire placenta was collected into a clean bucket and carried to a sampling station mounted within the barn. After weighing, the placenta was extended in a clean tray, and 5 cotyledons placed around the umbilicus insertion were selected for sampling and rinsed in 1× PBS before collection (Figure S1). Samples from each placenta were preserved using the following methods: 1. snap frozen (SF, *n* = 27): A small piece of each cotyledon was collected, and all pieces were chopped and mixed to obtain a combined sample. The tissue was placed into a 1.5 mL cryogenic tube and immediately frozen in liquid nitrogen. Samples were stored in liquid nitrogen for 6 weeks and then kept at −80 °C for about one month until further processing. 2. RNAlater^®^ (LTR, *n* = 27): A small piece was cut from the same cotyledons as in the SF sample. The five pieces (0.3 cm^3^ approximately) were placed into a 5 mL RNase-free tube with 4 mL of RNAlater^®^ (#AM721, Thermofisher, Waltham, MA, USA). Tubes were maintained at room temperature for 2 weeks, then at 4 °C for a month, and stored at −20 °C until further processing. RNAlater^®^ was not removed from the samples before freezing. It is important to mention that just cotyledon tissue was collected because, due to the structure of the sheep placenta, the caruncular tissue, which is part of the maternal uterine wall, is not expelled when the postpartum placenta is delivered.

The preservation protocols used in SF and LTR samples were adjusted to the working possibilities of our field conditions, which did not provide access to refrigerators or freezers during the lambing period (2 weeks) since there is no electricity connection in the research station, which is in the middle of Chilean Patagonia. Hence, it was not possible to preserve the LTR samples at cooling or freezer temperatures during that period of time. Immediately after finishing the tissue collection period, all samples were moved to our temporal storage station (1 h drive from the research station). At this time, SF samples were maintained in liquid nitrogen, and LTR samples were stored at 4 °C, which was the only cooling alternative available.

### 2.3. RNA Extraction

Approximately a month after collection, samples were shipped for RNA extraction and analysis from the Regional Research Center INIA Kampenaike in Punta Arenas, Magallanes Region, Chile, to the Applied Morphology Laboratory, Facultad de Ciencias Veterinarias of Universidad Austral de Chile (UACh), Valdivia, Los Ríos Region, Chile. The SF and LTR samples were shipped on independent Styrofoam boxes containing dry ice and ice, respectively. All samples were subject to the same shipping method, which included a 2.5 h flight followed by a 1.5 h drive to reach their final destination. Upon arrival, SF and LTR samples were immediately stored at −80 °C and −20 °C, respectively. Samples from both preservation methods were processed in parallel using the same protocol for RNA extraction. Briefly, all surfaces and implements were cleaned with an anti-RNAse solution (RNase*Zap*^®^ #AM9722, Invitrogen, Waltham, MA, USA). A portion of approximately 50 to 100 mg of tissue was collected and processed using an RNA extraction kit according to the manufacturer’s recommendations and adding the optional treatment with DNases included in the kit (Quick-RNA mini prep^®^ #R1054, Zymo Research, Irvine, CA, USA). The specific weight of each sample was not recorded in order to avoid thawing of the tissue. All samples were eluted in 50 uL of ultrapure RNAse-free water.

### 2.4. RNA Quantity and Quality Indicators

Concentration (ng/µL), A260/280 ratio, and RNA quality number (RQN) were used for quantity and quality assessment in SF and LTR samples. Concentration and A260/280 ratio were measured using a NanoDrop^®^ (ND-LITE Thermofisher, Waltham, MA, USA). RQN was assessed using a Fragment Analyzer^TM^ Automated CE system (Advanced Analytical, North Brunswick, NJ, USA). An A260/280 value between 1.8 and 2.2 was considered an ideal indicator for purity [27], while an RQN of 7 or above (scale of 1 to 10) was considered ideal for RNA integrity [12].

### 2.5. Data Analysis

Data were analyzed using the statistical software R (R version 4.3.0). Normality was evaluated using the Shapiro–Wilk test. Treatments were compared using a matched pairs analysis for concentration, A260/280 ratio, and RQN. Within each treatment, a correlation analysis was applied between all the described variables and the time of placental expulsion. Statistical significance was defined at *p* < 0.05. Data are presented as mean ± standard error of the mean (SEM).

## 3. Results

The complete dataset supporting the results described in this manuscript can be seen in Tables S1–S3 of the Supplementary Materials.

### 3.1. RNA Concentration and Purity

**Figure 1:**
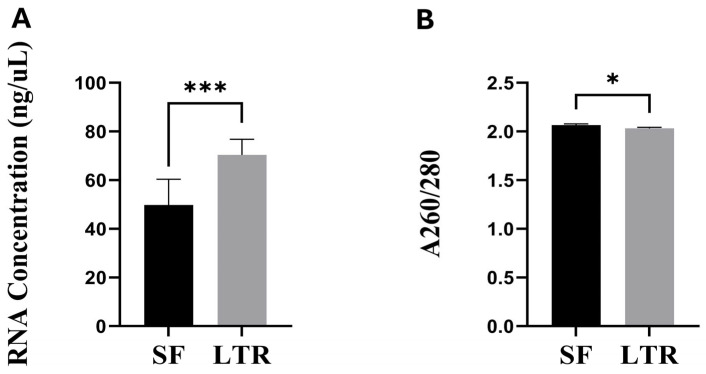
RNA concentrations (**A**) and A260/280 ratio (**B**) in naturally expelled ovine placental samples processed by snap frozen (SF, *n* = 27) and RNAlater^®^ (LTR, *n* = 27) preservation methods (*** = *p* < 0.001, * = *p* < 0.05).

The average RNA concentration was higher (*p* < 0.001) in LTR (70.39 ± 6.3 ng/µL) than in SF (49.77 ± 10.5 ng/µL) treatment (Figure 1A). The A260/280 ratio was slightly but significantly higher (*p* = 0.045) in SF (2.06 ± 0.01) than in LTR (2.03 ± 0.01) samples, respectively (Figure 1B).

### 3.2. RNA Integrity

**Figure 2:**
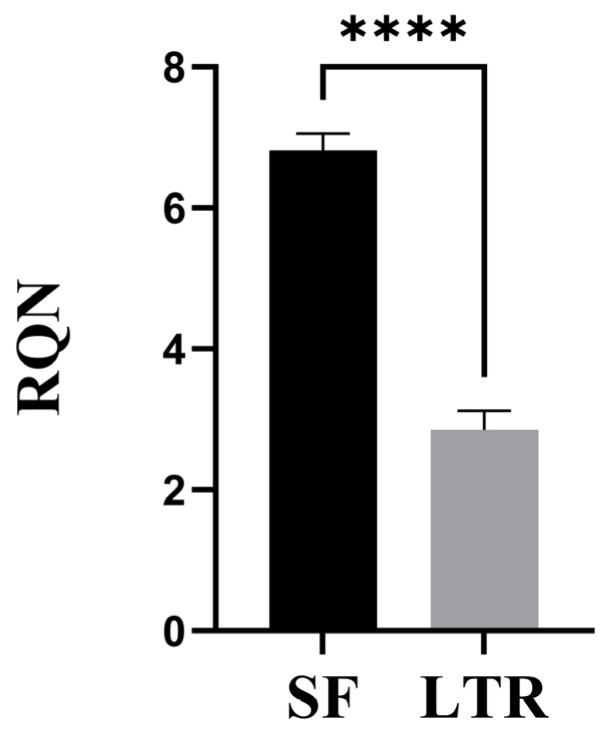
RQN values in naturally expelled ovine placental samples processed by snap frozen (SF, *n* = 27) and RNAlater^®^ (LTR, *n* = 27) preservation methods (**** = *p* < 0.0001).

There was a significant effect of the preservation method on RNA integrity. The RQN value was higher (*p* < 0.001) in SF (6.81 ± 0.24) than in LTR (2.84 ± 0.24) samples (Figure 2). In the SF group, 56% of the samples had RQN > 7, 89% had RQN > 6, and just 11% of the samples had RQN < 6. On the contrary, 100% of the LTR samples had RQN < 7, and 96% showed RQN < 6, with most of them being under 4. Just one sample had an exceptionally high RQN for the group (6.4).

### 3.3. Time of Placental Expulsion and Correlation to RNA Quantity and Quality Indicators

Average placental expulsion time was 3.18 ± 0.14 h, with a variation between 2 h and 5 h for the fastest and slowest delivery times, respectively. There were no significant correlations between time of placental expulsion and RNA concentration, purity, and integrity for SF or LTR samples (Table 1).

## 4. Discussion

This study aimed to evaluate the feasibility of extracting high quality RNA from naturally delivered placenta in sheep, to compare two preservation methods in order to identify the most adequate protocol for further transcriptomic analysis, and to evaluate if variation in placental expulsion timing has an impact on RNA quality and quantity parameters. Major findings are that RNA can be extracted from expelled ovine placental tissue, regardless of the preservation method. However, RNA integrity is higher in the tissue preserved by the snap frozen method. Another very interesting finding is that variation in timing for natural placental delivery does not affect RNA quantity and quality indicators.

Our results indicate that the concentration of RNA was higher in the LTR than in the SF treatments. This may be due to actual variation between methods, but it could also be an artifact. A precise amount of tissue was harder to obtain from SF than LTR samples, given the difficulty of cutting the sample without letting it thaw. As a result, it is possible that bigger pieces of tissue were obtained from SF than LTR samples, thus creating an unintended artifact effect in RNA concentration, since all samples were eluted in the same final volume. As the individual weight of each SF and LTR sample was not recorded, it was not possible to correct the final RNA concentration by sample weight. Hence, even when there are significant differences in RNA concentration between methods, we cannot assume that one method is better than the other in this aspect. Regardless, RNA concentration is less relevant than other parameters, such as RNA integrity, when performing transcriptomics analysis [28], so it is not the most relevant indicator to recommend one preservation method over the other. In terms of purity, even when the A260/280 ratio was slightly higher in SF than in LTR samples, both groups showed average values above 2, which are in the desired range to consider the extracted RNA to be free of contaminants such as proteins or other solvents derived from the extraction protocol [29].

The most relevant difference between the two methods was in RNA quality, which was considerably better in the SF group, at least under the protocol and conditions in which this study was performed. As explained earlier, the quality of RNA is an essential requirement for successful transcriptomics studies. For example, Huang et al. (2013) [30] showed that degraded RNA differentially affected the expression of endogenous reference genes in the human placenta. Other studies have also reported that, as RIN values decreased, the expression of genes in human placenta appeared falsely increased [31]. To our knowledge, this is the first report indicating that it is possible to obtain high quality RNA from naturally expelled ovine placentas. We expect that this will serve as a basis for other research groups to consider the application of this non-invasive sampling approach as an alternative to comply with the refinement principle of animal use in research.

Regardless of our results, we cannot obviate the possibility that RNAlater^®^ could provide better results using a different protocol. The fabricant recommends different alternatives to preserve tissue using RNAlater^®^ [32], which includes the one used in this study, except for the fact that we left the submerged samples at room temperature for two weeks. The reasons for this choice have been stated before and are related to the field conditions in which the study was conducted, which did not allow access to freezers or refrigerators during the lambing period, which lasted for 2 weeks. We found severe RNA degradation when using RNAlater^®^ as the preservation method under the described conditions, but we cannot rule out that RNA degradation could have been avoided if using RNAlater^®^ under a different protocol. However, other studies report similar results, indicating that better preservation of tissue is achieved using the snap frozen technique [33], even when leaving samples in RNAlater^®^ at 4 °C for just one day, and then freezing them, which fully complies with the fabricant recommendations.

By contrast, a time course study analysis of RNA quality in the human placenta found that preservation in RNAlater^®^ yielded a higher RIN than the snap frozen technique in long-term stored samples, while there were no differences between methods when stored for a short time before transcriptomics analysis [14]. However, in this study, samples preserved in RNAlater^®^ were immediately frozen. Similarly, another study reported that RIN values were higher in human placental tissue collected up to two hours after delivery [34] when preserving them in RNAlater^®^ (4 °C overnight, −80 °C for long-term storage) compared to snap-frozen technique. Currently, the literature for the human placenta is still not clear on which preservation method is more suitable for the extraction of high-quality RNA [3], and to our knowledge, a guidance in this regard is inexistent for the ovine placenta. Most likely, the best preservation method would be dependent on the particular conditions in which each experiment is conducted and the resources that the investigator has access to.

Regardless of the preservation method, the literature usually suggests that placental samples must be collected as soon as possible [3], as this tissue is high in RNase activity [15], and it also rapidly degrades after parturition. In naturally expelled ovine placenta, the time of collection is a major concern because this organ may take up to 6 h to be delivered [23]. There is also considerable inter-animal variability in the time of placental expulsion, which in the present study ranged between 2 and 5 h. Although successful RNA extraction from the postpartum placenta has been described in the bovine [35] and equine [36], in those studies samples, were collected trans cervically at 30 min and 2 h postpartum, respectively. Here, we largely exceed those times, and the inter-animal variation was impossible to control by the investigator, being critical as it may differentially impact RNA quality. For example, a study in the human placenta described that RNA RIN has an inverse correlation with sampling delay [31], but in this case, placental collection was delayed while the organ was already outside the maternal body. In our study, the placenta remained inside the body of the animal and was immediately sampled once expelled, so the sampling delay period did not occur under environmental conditions. This may be associated with the fact that we did not find a significant correlation between the time of placental expulsion and RNA concentration, A260/280 ratio or, most importantly, RQN in both SF and LTR samples. This is also supported by other studies that have found no association between a 24 h sampling delay and RNA yield or quality in postmortem reproductive bovine tissues [37]. Research from the human placenta also states that storing placental tissue a 4 °C up to 48 h does not affect RNA quality [38]. The findings of those studies, and the ones of the present research, are relevant to support the applicability of post-delivery ovine placental collection, which opens possibilities to perform transcriptomics studies in an organ that is hard to collect without applying invasive procedures or euthanasia.

Although time for placental expulsion does not affect RNA quality, it is unknown if this could have an effect on gene expression in the event that gene expression patterns change over time from parturition to natural placental delivery. This has not been evaluated in the ovine placenta, and it warrants further investigation. However, data from studies in bovine reproductive tissue indicate that there is no impact on the expression of genes such as β-actin, GAPDH, and transforming growth factor β in samples collected up to 96 h postmortem [37]. Also, studies from the human placenta indicate that meaningful gene expression analysis can be performed from RNA extracted from placentas stored at 4 °C or even at room temperature for up to 48 h, as long as RNA integrity is preserved [38]. These results indicate that the time of tissue collection does not affect gene expression in samples with preserved RNA integrity. However, a recent study from the human placenta suggests that some mRNA transcripts may change as placental collection time increases, regardless of RNA quality [39]. This is an issue that requires further investigation in samples from naturally expelled sheep placenta, as available literature is still contradictory and mostly from humans.

Other limitations of obtaining RNA from the expelled ovine placenta, regardless of sample quality, are associated with the fact that this method allows for the collection of cotyledonary tissue but not caruncular tissue, which is not expulsed. Additionally, since gestation is not intervened, this method does not allow for performing time-course studies of placental gene expression throughout pregnancy. The investigator must evaluate how much these limitations may impact the required data according to the purpose of the research, while also considering that the method validated through this research aligns with the refinement principle for animals used in research as it significantly reduces the invasiveness of the sampling method [25,26].

## 5. Conclusions

We found that the preservation in liquid nitrogen (snap frozen) immediately after natural placental expulsion is an adequate method to obtain high-quality RNA from the ovine placenta when working under field conditions. This is valuable information for placental biologists working with the sheep model, as it demonstrates the possibility of obtaining tissue samples without having to apply non—recovery or complex surgical methods. It also allows for follow-up studies in the offspring, which is most likely lost in the aforementioned approaches. We also highlight the fact that the time from parturition to placental expulsion does not correlate with RNA quantity or quality, eliminating this issue as a factor of concern. Regardless of the recognized limitations of the method, this work offers an alternative to comply with the refinement principle for animal use in research.

## Figures and Tables

**Table 1:** Correlation coefficients between placental expulsion time and RNA quantity, purity, and quality indicators by preservation method.

Preservation Method	Indicator	Correlation Coefficient (r^2^)	*p*-Value
Snap Frozen (SF)	Concentration (ng/µL)	0.010	0.6125
	A260/280 Ratio	0.063	0.2065
	RNA Quality Number (RQN)	0.002	0.7874
RNA*later*^®^ (LTR)	Concentration (ng/µL)	0.084	0.1405
	A260/280 Ratio	0.002	0.8058
	RNA Quality Number (RQN)	0.050	0.2615

## Data Availability

Dataset available on Supplementary Materials.

## Supplementary Materials

The following supporting information can be downloaded at: https://www.mdpi.com/article/10.3390/ani15060786/s1, Figure S1: Illustrative image of a delivered placenta from the study and sites of tissue collection. Table S1: RNA concentration (µg/µL) in delivered ovine placental samples preserved using snap frozen (SF) and RNA*later*^®^ (LTR). Table S2: Ratio A260/280 in RNA from delivered ovine placental samples preserved using snap frozen (SF) and RNA*later*^®^ methods. Table S3: RNA Quality Number (RQN) in delivered ovine placental samples preserved using snap frozen (SF) and RNA*later*^®^ (LTR).

## Abbreviations

The following abbreviations are used in this manuscript:

RNA	Ribonucleic Acid
GD	Gestational Day
SF	Snap-Frozen treatment
LTR	RNAlater^®^ treatment
RQN	RNA Quality Number
RIN	RNA Integrity Number

## References

[1] Sammin D., Markey B., Bassett H., Buxton D. (2009). The ovine placenta and placentitis-A review. Vet. Microbiol..

[2] Tanner A.R., Kennedy V.C., Lynch C.S., Hord T.K., Winger Q.A., Rozance P.J., Anthony R.V. (2022). In vivo investigation of ruminant placenta function and physiology-A review. J. Anim. Sci..

[3] Arthurs A.L., McCullough D., Williamson J.M., Jankovic-Karasoulos T., Smith M.D., Roberts C.T. (2023). Factors influencing RNA yield from placenta tissue. Placenta.

[4] Steinhauser C.B., Lambo C.A., Askelson K., Burns G.W., Behura S.K., Spencer T.E., Bazer F.W., Satterfield M.C. (2021). Placental transcriptome adaptations to maternal nutrient restriction in sheep. Int. J. Mol. Sci..

[5] Barry J.S., Anthony R.V. (2008). The pregnant sheep as a model for human pregnancy. Theriogenology.

[6] Banstola A., Reynolds J.N.J. (2022). The Sheep as a Large Animal Model for the Investigation and Treatment of Human Disorders. Biology.

[7] Leiser R., Krebs C., Ebert B., Dantzer V. (1997). Placental vascular corrosion cast studies: A comparison between ruminants and humans. Microsc. Res. Tech..

[8] Carter A.M. (2018). Recent advances in understanding evolution of the placenta: Insights from transcriptomics. F1000Research.

[9] Miller C.L., Diglisic S., Leister F., Webster M., Yolken R.H. (2004). Evaluating RNA status for RT-PCR in extracts of postmortem human brain tissue. Biotechniques.

[10] Sidova M., Tomankova S., Abaffy P., Kubista M., Sindelka R. (2015). Effects of post-mortem and physical degradation on RNA integrity and quality. Biomol. Detect. Quantif..

[11] Schroeder A., Mueller O., Stocker S., Salowsky R., Leiber M., Gassmann M., Lightfoot S., Menzel W., Granzow M., Ragg T. (2006). The RIN: An RNA integrity number for assigning integrity values to RNA measurements. BMC Mol. Biol..

[12] Roume H., Heintz-Buschart A., Muller E.E.L., Wilmes P. (2013). Sequential isolation of metabolites, RNA, DNA, and proteins from the same unique sample. Methods in Enzymology.

[13] Opitz L., Salinas-Riester G., Grade M., Jung K., Jo P., Emons G., Ghadimi B.M., Beißbarth T., Gaedcke J. (2010). Impact of RNA degradation on gene expression profiling. BMC Med. Genom..

[14] Martin N.M., Cooke K.M., Radford C.C., Perley L.E., Silasi M., Flannery C.A. (2017). Time course analysis of RNA quality in placenta preserved by RNA later or flash freezing. Am. J. Reprod. Immunol..

[15] Haimov-Kochman R., Fisher S.J., Winn V.D. (2006). Modification of the Standard Trizol-Based Technique Improves the Integrity of RNA Isolated from RNase-Rich Placental Tissue. Clin. Chem..

[16] Desai N.A., Shankar V. (2003). Single-strand-specific nucleases. FEMS Microbiol. Rev..

[17] Peirson S.N., Butler J.N. (2007). RNA extraction from mammalian tissues. Methods Mol. Biol..

[18] Horcajo P., Ortega-Mora L.M., Benavides J., Sánchez-Sánchez R., Amieva R., Collantes-Fernández E., Pastor-Fernández I. (2023). Ovine placental explants: A new ex vivo model to study host-pathogen interactions in reproductive pathogens. Theriogenology.

[19] Erichsen C., Heiser A., Haack N., Maclean P., Dwyer C.M., McCoard S. (2024). Increasing the Understanding of Nutrient Transport Capacity of the Ovine Placentome. Animals.

[20] Tong W., Allison B.J., Brain K.L., Patey O.V., Niu Y., Botting K.J., Ford S.G., Garrud T.A., Wooding P.F.B., Lyu Q. (2025). Placental mitochondrial metabolic adaptation maintains cellular energy balance in pregnancy complicated by gestational hypoxia. J. Physiol..

[21] Lambo C.A., Edwards A.K., Bazer F.W., Dunlap K., Satterfield M.C. (2020). Development of a surgical procedure for removal of a placentome from a pregnant ewe during gestation. J. Anim. Sci. Biotechnol..

[22] Persson K., Rodriguez Perez C., Louis-Maerten E., Müller N., Shaw D. (2025). “Killing in the Name of 3R?” The Ethics of Death in Animal Research. J. Agric. Environ. Ethics.

[23] Fthenakis G.C., Leontides L.S., Amiridis G.S., Saratsis P. (2000). Incidence risk and clinical features of retention of foetal membranes in ewes in 28 flocks in southern Greece. Prev. Vet. Med..

[24] Auer H., Mobley J., Ayers L., Bowen J., Chuaqui R., Johnson L., Livolsi V., Lubensky I., McGarvey D., Monovich L. (2014). effects of frozen tissue storage conditions on the integrity of RNA and protein. Biotech. Histochem..

[25] Costa J., Mackay R., de Aguiar Greca S.-C., Corti A., Silva E., Karteris E., Ahluwalia A. (2021). The role of the 3rs for understanding and modeling the human placenta. J. Clin. Med..

[26] MacArthur Clark J. (2018). The 3Rs in research: A contemporary approach to replacement, reduction and refinement. Br. J. Nutr..

[27] Lucena-Aguilar G., Sánchez-López A.M., Barberán-Aceituno C., Carrillo-Ávila J.A., López-Guerrero J.A., Aguilar-Quesada R. (2016). DNA Source Selection for Downstream Applications Based on DNA Quality Indicators Analysis. Biopreserv. Biobank..

[28] Vermeulen J., De Preter K., Lefever S., Nuytens J., De Vloed F., Derveaux S., Hellemans J., Speleman F., Vandesompele J. (2011). Measurable impact of RNA quality on gene expression results from quantitative PCR. Nucleic. Acids Res..

[29] Shah S.G., Rashid M., Verma T., Ludbe M., Khade B., Gera P.B., Gupta S. (2019). Establishing a correlation between RIN and A260/280 along with the multivariate evaluation of factors affecting the quality of RNA in cryopreserved cancer bio-specimen. Cell Tissue Bank..

[30] Huang X., Baumann M., Nikitina L., Wenger F., Surbek D., Körner M., Albrecht C. (2013). RNA degradation differentially affects quantitative mRNA measurements of endogenous reference genes in human placenta. Placenta.

[31] Jobarteh M.L., Moore S.E., Kennedy C., Gambling L., McArdle H.J. (2014). The effect of delay in collection and processing on RNA integrity in human placenta: Experiences from rural Africa. Placenta.

[32] ThermoFisher Scientific RNAlater Tissue Collection: Stabilization Solution Protocol. https://tools.thermofisher.com/content/sfs/manuals/cms_056069.pdf.

[33] Ozkan H., Kerman E. (2020). Comparative Evaluation of RNAlater Solution and Snap Frozen Methods for Gene Expression Studies in Different Tissues. Rev. Rom. Med. Lab..

[34] Wolfe L., Thiagarajan R., Boscolo F., Taché V., Coleman R., Kim J., Kwan W., Loring J., Parast M., Laurent L. (2014). Banking placental tissue: An optimized collection procedure for genome-wide analysis of nucleic acids. Placenta.

[35] Moradi M., Zhandi M., Sharafi M., Akbari A., Atrabi M.J., Totonchi M. (2022). Gene expression profile of placentomes and clinical parameters in the cows with retained placenta. BMC Genom..

[36] Jaworska J., Janowski T. (2019). Expression of proinflammatory cytokines IL-1β, IL-6 and TNFα in the retained placenta of mares. Theriogenology.

[37] Fitzpatrick R., Casey O.M., Morris D., Smith T., Powell R., Sreenan J.M. (2002). Postmortem stability of RNA isolated from bovine reproductive tissues. Biochim. Biophys. Acta (BBA)-Gene Struct. Expr..

[38] Fajardy I., Moitrot E., Vambergue A., Vandersippe-Millot M., Deruelle P., Rousseaux J. (2009). Time course analysis of RNA stability in human placenta. BMC Mol. Biol..

[39] Freedman A.A., Smart B.P., Keenan-Devlin L.S., Romero J., Franklin A., Borders A., Ernst L.M., Miller G.E. (2021). Time-dependent changes in placental mRNA expression after delivery due to delayed specimen collection. Am. J. Reprod. Immunol..

