# Supplementary material for: Comparison Between Two Methodologies of Sample Preservation for RNA Extraction in Naturally Delivered Ovine Placenta
Source: Animals (Basel). 2025 Mar 10;15(6):786. doi: 10.3390/ani15060786 (PMC11939649; doi:10.3390/ani15060786)
Supplement: Supplementary file 1 [file animals-15-00786-s001.zip › animals-3481289-supplementary.pdf]

Figure S1. Illustrative image of a delivered placenta from the study and sites of tissue collection. The white arrow indicates the umbilicus insertion. Yellow arrows indicate the 5 sampled cotyledons, placed closest to the umbiliculs insertion.

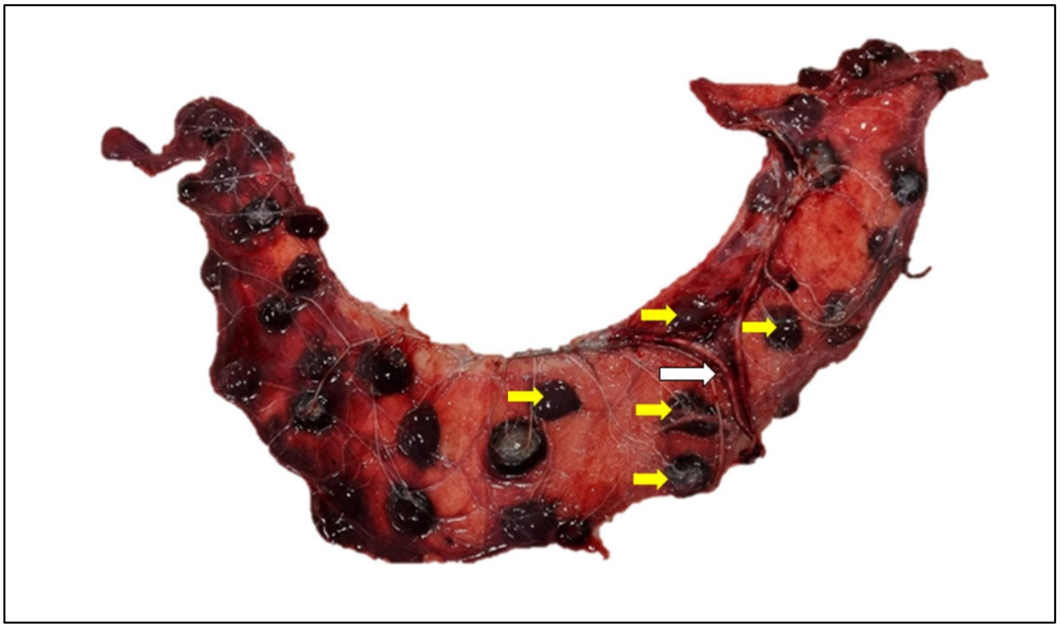

Table S1. RNA concentration (ng/uL) in delivered ovine placental samples preserved using snap frozen (SF) and RNA*later*® methods.

	RNA Concentration (ng/uL)	
**Sample ID**	**Snap Frozen (SF)**	**RNA*later*® (LTR)**
37958	28.6	154.6
38046	16.5	56.4
38345	66.7	77.1
38532	73.2	46.5
38654	84.5	101.4
43190	265.9	85.2
43324	31.6	34.3
42965	20.0	87.7
42826	35.8	115.3
41981	76.3	101.2
38047	18.9	76.7
42869	19.4	75.6
42912	12.4	75.8
43047	27.5	40.8
43631	16.8	71.8
43667	30.6	97.1
37957	39.7	37.1
38346	29.5	127.4
38634	22.3	70.6
42356	23.6	28.2
42404	54.8	78.6
42814	31.9	88.4
42846	49.5	39.6
42851	18.7	34.3
42858	32.6	28.4
43001	35.3	45.8
43593	181.2	24.8

Table S2. Ratio A260/280 in RNA from delivered ovine placental samples preserved using snap frozen (SF) and RNA*later*® methods.

	Ratio A260/280	
**Sample ID**	**Snap Frozen (SF)**	**RNA*later*® (LTR)**
37958	2.08	2.06
38046	1.97	2.05
38345	1.97	2.02
38532	2.09	2.04
38654	2.11	1.99
43190	2.10	1.95
43324	2.20	1.99
42965	2.09	2.12
42826	2.09	1.95
41981	2.09	1.93
38047	2.07	2.02
42869	2.02	2.08
42912	2.01	2.07
43047	2.05	2.11
43631	2.02	2.10
43667	2.13	2.10
37957	2.10	1.97
38346	2.08	2.04
38634	1.97	1.95
42356	2.04	1.94
42404	2.03	1.98
42814	2.01	2.07
42846	2.04	2.03
42851	2.01	2.05
42858	2.11	2.06
43001	2.19	2.06
43593	2.11	2.13

Table S3. RNA Quality Number (RQN) in delivered ovine placental samples preserved using snap frozen (SF) and RNA*later*® methods.

	RNA Quality Number (RQN)	
**Sample ID**	**Snap Frozen (SF)**	**RNA*later*® (LTR)**
37958	7.1	2.0
38046	8.6	2.7
38345	7.3	3.3
38532	6.5	1.2
38654	6.2	3.7
43190	7.0	3.3
43324	7.9	3.2
42965	8.6	4.0
42826	7.1	3.5
41981	7.3	3.1
38047	7.3	4.5
42869	8.1	6.5
42912	6.5	5.5
43047	6.1	5.2
43631	7.3	3.0
43667	7.0	4.0
37957	7.8	1.4
38346	7.1	2.8
38634	2.6	1.8
42356	6.8	1.8
42404	6.5	1.4
42814	6.6	1.5
42846	6.1	2.5
42851	5.4	1.3
42858	6.4	1.2
43001	4.4	1.2
43593	8.4	1.1
